# Feasibility Tests on Concrete with Very-High-Volume Supplementary Cementitious Materials

**DOI:** 10.1155/2014/406324

**Published:** 2014-08-06

**Authors:** Keun-Hyeok Yang, Yong-Su Jeon

**Affiliations:** ^1^Department of Plant Architectural Engineering, Kyonggi University, Suwon-si, Gyeonggi-do 443-760, Republic of Korea; ^2^Department of Architectural Engineering, Graduate School, Kyonggi University, Suwon-si, Gyeonggi-do 443-760, Republic of Korea

## Abstract

The objective of this study is to examine the compressive strength and durability of very high-volume SCM concrete. The prepared 36 concrete specimens were classified into two groups according to their designed 28-day compressive strength. For the high-volume SCM, the FA level was fixed at a weight ratio of 0.4 and the GGBS level varied between the weight ratio of 0.3 and 0.5, which resulted in 70–90% replacement of OPC. To enhance the compressive strength of very high-volume SCM concrete at an early age, the unit water content was controlled to be less than 150 kg/m^3^, and a specially modified polycarboxylate-based water-reducing agent was added. Test results showed that as SCM ratio (*R*_SCM_) increased, the strength gain ratio at an early age relative to the 28-day strength tended to decrease, whereas that at a long-term age increased up to *R*_SCM_ of 0.8, beyond which it decreased. In addition, the beneficial effect of SCMs on the freezing-and-thawing and chloride resistances of the concrete decreased at *R*_SCM_ of 0.9. Hence, it is recommended that *R*_SCM_ needs to be restricted to less than 0.8–0.85 in order to obtain a consistent positive influence on the compressive strength and durability of SCM concrete.

## 1. Introduction

Ordinary Portland cement (OPC), an essential construction material, has contributed substantially to building and infrastructure development. However, since the late 1990s the concrete industries have exerted considerable effort and made investments to minimize the use of OPC, partly because of serious worldwide issue to reduce greenhouse gas emissions. It is generally estimated that the production of one ton of OPC consumes approximately 2.8 tons of raw materials such as limestone and coal and that it releases about 0.7–0.95 tons of carbon dioxide (CO_2_) into the Earth's atmosphere from the decarbonation of lime in the kiln and the combustion of fuels [1, 2]. Because of the high CO_2_ inventory of OPC, the annual emission of greenhouse gases from the worldwide production of OPC is estimated to be approximately 1.35 billion tons [3]. Furthermore, the average electricity consumption in cement manufacturing is given as 106 kWh/ton, which is equivalent to approximately 1.2 GJ/ton in primary energy [3]. For these reasons, a stronger effort is required for the development of an alternative practical concrete technology that ensures low CO_2_ emissions.

The use of high-volume supplementary cementitious materials (SCMs) as partial replacement for OPC in concrete has become increasingly attractive for the development of sustainable construction materials with low CO_2_ emissions. As a result, the practical application of by-products such as fly ash (FA) and ground granulated blast-furnace slag (GGBS) as SCMs has gradually increased in the construction industry because of their environmentally beneficial recycling effect and remarkably low CO_2_ inventory [4]. Furthermore, the appropriate addition of SCMs in place of OPC can improve concrete properties as follows [5–8]. (1) The pozzolanic activity of SCMs is effective for forming a denser matrix, leading to higher strength (especially at a long-term age) and better durability of the concrete; namely, the pozzolanic activity improves the impermeability of the concrete through the formation of calcium silicate hydrate (CSH) and calcium aluminate hydrate (CAH) gels. (2) FA with spherically shaped particles improves the workability of fresh concrete, which reduces the demand for water for targeted workability and leads to reduced bleeding and less shrinkage deformation of the concrete. (3) The temperature increase during cement hydration is controlled, which helps reduce cracking in mass concrete at early ages. To attain these positive effects, the typical individual limitation for OPC replacement is commonly estimated to be 15–20% for FA and 40–50% for GGBS [9]. On the other hand, it has been commonly pointed out [10, 11] that a large amount of SCMs is not helpful in improving the workability of concrete to any considerable extent because of their low density. Furthermore, the longer curing time owing to their slow pozzolanic reaction which converts soluble alkali into a more stable CSH gel requires longer curing time needed to gain targeted concrete strength. This indicates that a relatively higher strength gain at an early age is one of the essential considerations for the practical use of high-volume SCM concrete.

Malhotra et al. [10–12] did pioneering work on high-volume FA concrete and conducted extensive studies to establish and improve the characteristics of concrete containing large amounts of SCMs. Mahmoud et al. [13] showed that concrete mixes made with a ternary binder that incorporated both FA and GGBS have an advantage in terms of early strength development over concretes with FA alone. Huang et al. [14] confirmed the feasibility of using up to 80% Class F of FA as an OPC replacement in concrete if rational mixture proportions are provided. Chen et al. [6] proposed that the amount of cement paste and the water content need to be minimized in order to obtain good quality concrete containing a high volume of FA and GGBS. Lee and Wu [15] reported that FA with a high loss-on-ignition (LOI) value has an adverse influence on the strength and durability of concrete. Yazıcı [16] demonstrated that the chloride-ion penetration depth of concrete decreased with the increase in substitution level of FA up to 30%, beyond which it is marginally affected by the FA content. Overall, from a review of recent experimental observations, it can be concluded that the extent of improvement of the strength and durability of high-volume SCM concrete depends on the mixture proportions of each ingredient for concrete and the chemical composition and the quality of the SCMs. Moreover, the optimization of high-volume SCMs needs to be qualified for required specification in the intended application of concrete.

The objective of the present study is to examine the practical feasibility of producing very-high-volume SCM concrete (incorporating FA and GGBS) with relatively good strength gain at an early age. A total of 36 high-volume SCM concrete mixes with different mixture proportions were prepared according to designed concrete compressive strengths of 24 MPa (Group I) and 30 MPa (Group II). As a partial replacement for OPC, the weight ratio of FA (*R*_*F*_) was fixed at 0.4, whereas that of GGBS (*R*_*G*_) varied between 0.3 and 0.5; as a result, 70–90% of the OPC was replaced with FA and GGBS. Concrete mixes with *R*_*F*_ of 0.25 and *R*_*G*_ of 0.15 were also prepared as control specimens in each group. To achieve good strength gain, especially at an early age, the unit water content was controlled to be less than 150 kg/m^3^, and a polycarboxylate-based water-reducing agent was added after being specially modified through the adjustment of the amount of polyethylene glycol alkyl ether and the addition of an amine. Simple equations to predict strength development of the very-high-volume SCM concrete samples are proposed based on the nonlinear multiple regression analysis of the measured results. Four very-high-volume SCM specimens, together with the companion control mixes, were selected in order to examine their durability under the following environments: repeated freezing and thawing, chloride penetration, and sulfate attack.

## 2. Experimental Program

### 2.1. Materials

OPC (ASTM Type I) was partially replaced with commercially available FA and GGBS powders, which produces a ternary-type binder. The chemical compositions of these materials were determined by X-ray fluorescence (XRF) analysis and the results are given in Table 1. The FA had low calcium oxide (CaO) and a silicon oxide (SiO_2_)-to-aluminum oxide (Al_2_O_3_) ratio by mass of 1.91, which belongs to Class F of ASTM C618 [17]. The LOI and 28-day activity coefficient of FA were 0.89% and 92%, respectively. GGBS conforming to ASTM C989 [17] had high CaO and a SiO_2_-to-Al_2_O_3_ ratio by mass of 2.29, which is very similar to that of OPC. The basicity of GGBS calculated from the chemical composition was 1.94. The specific gravity and specific surface area, respectively, were 3.15 and 3466 cm^2^/g for OPC, 2.23 and 3720 cm^2^/g for FA, and 2.91 and 4497 cm^2^/g for GGBS.

Locally available natural sand with a maximum particle size of 5 mm and crushed granite with a maximum particle size of 25 mm were used for fine aggregates and coarse aggregates, respectively. The specific gravity and water absorption were 2.61 and 1.16%, respectively, for fine aggregate and 2.62 and 1.78% for coarse aggregate, as given in Table 2. The moduli of fineness of the fine and coarse aggregates were 2.83 and 7.05, respectively.

**Figure 1:**
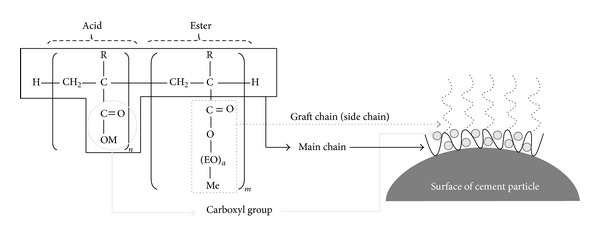
Molecular structure of polycarboxylate-based water-reducing agent used.

To maintain good workability of the concrete at lower unit water content, the molecular structure of a polycarboxylate-based water-reducing agent was specially modified as follows. (1) The degree of polymerization of the main chain in the acryl acid-type polycarboxylate polymer was reduced by a factor of 10 (see Figure 1). (2) The molecular weight of a polyethylene glycol mono-alkyl ether monomer was increased to 2000 in order to increase the length of the graft chain in the polycarboxylate polymer. The decreased length of the main chain and increased length of the graft chain are effective for enhancing the dispersibility of polycarboxylate polymers in cement pastes. Furthermore, to obtain an increase in the strength of the concrete at an early age, an amine was added to the modified polycarboxylate polymer. It is known [18] that the addition of an amine is helpful in catalyzing the hydration reaction of cement at an early age because it accelerates the leaching rate of Ca^2+^ and OH^−^ ions from the mineral compositions of the cement. From previous tests [19], the optimum dosage of the amine was determined to be 3% of the modified polycarboxylate polymer weight.

### 2.2. Specimens and Mixture Proportions

Table 3 shows the main mixture parameters for concrete specimens using FA and GGBS to achieve the targeted properties. All concrete mixes were classified into two groups according to the designed 28-day compressive strength (*f*_*cu*_) of 24 MPa (Group I) and 30 MPa (Group II). The selected test parameters in each group were as follows. (1) Two levels of the water content (*W*) were used, 140 kg/m^3^ and 150 kg/m^3^. (2) The unit binder content (*B*) for each water content level was varied as 310 kg/m^3^, 330 kg/m^3^, and 350 kg/m^3^ for Group I and 370 kg/m^3^, 390 kg/m^3^, and 410 kg/m^3^ for Group II; as a result, the water-to-binder ratios (*W*/*B*) for the Group I mixes were calculated to be 45.2%, 42.4%, and 40.0%, respectively, for *W* of 140 kg/m^3^ and 48.4%, 45.5%, and 42.9% for *W* of 150 kg/m^3^, while those in the Group II mixes were 37.8%, 35.9%, and 34.2% for *W* of 140 kg/m^3^ and 40.5%, 38.5%, and 36.6% for *W* of 150 kg/m^3^. (3) SCM level (*R*_SCM_) as a partial replacement for OPC was varied as 0.7, 0.8, and 0.9. At each *R*_SCM_, *R*_*F*_ was fixed to be 0.4, whereas *R*_*G*_ varied as 0.3, 0.4, and 0.5. The addition of FA as a partial replacement of OPC is favorable to the reduction of hydration heat of concrete but unfavorable to the strength development of concrete at an early age. Considering this fact, the present study selected *R*_*F*_ to be 0.4. The volumetric fine aggregate-to-total aggregate ratio (*S*/*a*) was designed to be 48% for *W* of 140 kg/m^3^ and 46% for *W* of 150 kg/m^3^. For comparison, a control mix with a typical *R*_SCM_ (*R*_*F*_ of 0.25 and *R*_*G*_ of 0.15) was also prepared for each group. Considering the demand increase trend on the use of SCM, the typical SCM concrete was selected for the control mix, instead of OPC concrete. From the practical mixture proportions of ready-mixed concrete batches, the unit water and binder contents determined for the control mixes were 184 kg/m^3^ and 342 kg/m^3^, respectively, for Group I and 165 kg/m^3^ and 400 kg/m^3^ for Group II. The targeted air content and initial slump of all concrete mixes were 4.5 ± 1.5% and 210 ± 25 mm, respectively. To meet the designed initial air content (*A*_*c*_) and slump (*S*_*i*_), an air entraining agent and the specially modified polycarboxylate-based high-range water-reducing agent were added, as given in Table 4. The state of moisture in aggregates was measured before the mix of concrete, and the surface water on aggregates was then reflected through the correction of the unit water content.

For easy recognition of test parameters, the concrete specimens were notated sequentially using the targeted compressive strength, water content, binder content, and SCM level as a partial replacement for OPC. For example, specimen I-140-310-0.7 indicates a concrete with *f*_*cu*_ of 24 MPa produced from the following mixture proportions: *W* of 140 kg/m^3^, *B* of 310 kg/m^3^, and *R*_SCM_ of 0.7 (*R*_*F*_ of 0.4 and *R*_*G*_ of 0.3). Concrete specimens denoted by I-C and II-C indicate the control concrete with a typical *R*_SCM_ value in each group.

### 2.3. Casting, Curing, and Testing

All concrete specimens were mixed using a twin forced mixing-type mixer with 0.35 m^3^ capacity. The initial slump (*S*_*i*_) and air content (*A*_*c*_) of fresh concrete were measured in accordance with the ASTM C143 and C231 provisions, respectively [17]. All specimens were cured under water with temperature of 23 ± 2°C until testing at a specified age. All steel molds were removed after aging for 36 h.

The compressive strength of the concrete was measured using cylindrical specimens of 100 mm in diameter and 200 mm high at ages of 3, 7, 28, 56, and 91 days in accordance with ASTM C39 [17]. The durability properties (freezing-and-thawing, chloride ion penetration, and sulfate resistances) were examined for the four selected very-high-volume SCM concrete mixes and two control mixes. All specimens used to measure the durability were demolded at an age of 1 day. The resistance to the freezing-and-thawing cycle of concrete was determined using 100 × 75 × 400 mm prisms in accordance with procedure A specified in ASTM C666 [17]. Prior to the rapid freezing-and-thawing test, the prism specimens were cured for 14 days and saturated in lime water for 48 h. With the start of tests, the relative dynamic modulus of elasticity was recorded at intervals of 30 cycles of freezing-and-thawing up to a maximum of 300 cycles. The resistance to chloride penetration was measured at ages of 28 and 91 days in accordance with a nonsteady-state migration test described in NT Build 492 [20]. Concrete cylinders (100 mm in diameter and 200 mm long) were sawn into disks with 50 mm thick. After vacuum saturation of the cylindrical test specimens in a Ca(OH)_2_ solution (4 g/L), an external electrical potential was applied axially across the specimen, forcing the chloride ions outside to migrate into the specimen. The catholyte solution was a 10% NaCl solution, whereas the anolyte solution was a 0.3 N NaOH solution. The penetration depth, measured from the visible white silver chloride precipitation at saturation ages of 28 and 91 days, was then converted into the chloride migration coefficient according to the procedure specified in NT Build 492. The sulfate resistance of the concrete was evaluated from the variations of compressive strength of the specimens saturated in a curing tank containing 5% sulfuric acid solution for 28 days.

## 3. Test Results and Discussion

### 3.1. Initial Slump and Air Content

The ratios of the modified polycarboxylate-based water-reducing agent (*R*_*SP*_) and air entraining agent (*R*_*A*_) to the total binder by weight used to achieve the target *S*_*i*_ and *A*_*c*_ are given in Table 4. In general, a greater amount of *R*_*A*_ was required for the very-high-volume SCM concrete mixes than for the companion control mixes, regardless of *W* and *R*_SCM_ values. The value of *R*_*A*_ was between 0.028% and 0.042% for Group I mixes and between 0.032% and 0.045% for Group II mixes, indicating that *A*_*c*_ of fresh concrete without the air-entraining agent was commonly lower in Group II mixes than in Group I mixes. To achieve the target compressive strength, a greater amount of binder was needed for the Group II mixes than for the Group I mixes at the same water content. This implies that increasing *B* at the same water content is accompanied by a decrease in the number of macrocapillaries and artificial air pores [9]. The specially modified polycarboxylate-based water-reducing agent was commonly added in the amount of 0.7–1.0% of the binder weight for the concrete mixes tested. The value of *R*_*SP*_ added to meet the targeted *S*_*i*_ was slightly higher for the Group II mixes than for the Group I mixes. This is attributed to the fact that *W*/*B* of the Group II mixes was lower than that of the Group I mixes. On the other hand, the value of *R*_*SP*_ tended to be independent of *R*_SCM_, indicating that the GGBS content has little influence on the workability of concrete [9].

### 3.2. Compressive Strength at 28 Days

**Figure 2:**
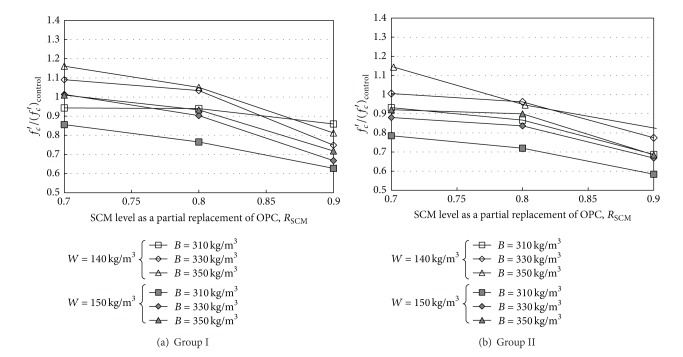
Relative 28-day strength of very-high-volume concrete as compared to control concrete.

Most concrete mixes with *W* of 140 kg/m^3^ met the targeted 28-day compressive strength (*f*_*cu*_), as given in Table 4. However, some specimens with *W* of 150 kg/m^3^ failed to achieve *f*_*cu*_, in particular, for the concrete with *R*_SCM_ of 0.9, and for the Group I concrete with *W*/*B* of 48.4% and the Group II concrete with *W*/*B* of 40.5%. As expected, the measured 28-day compressive strength (*f*_*c*_′) decreased with increasing *W*/*B* and *R*_SCM_. The ratio of *f*_*c*_′ of the very-high-volume SCM concretes relative to that of the control concrete is shown in Figure 2. The relative 28-day strength commonly decreased with increasing *R*_SCM_, indicating that the rate of the decrease was greater for Group II mixes than that for Group I mixes. All concrete mixes with *R*_SCM_ more than 0.8 developed lower *f*_*c*_′ than the control concrete. Furthermore, *f*_*c*_′ of the concrete with *W* of 150 kg/m^3^ was commonly lower by approximately 10% than that of the control concrete with *W* of 140 kg/m^3^, even at the same *W*/*B*, indicating that *f*_*c*_′ of very-high-volume SCM concrete is somewhat affected by *W*. Overall, to obtain a value of *f*_*c*_′ equivalent to that of a conventional concrete with a typical *R*_SCM_, very-high-volume SCM concrete should have *W*/*B* < 40% and *R*_SCM_ = 0.7.

In general, *f*_*c*_′ is taken to be inversely proportional to *W*/*B* and *A*_*c*_ [9]. Considering this fact, Yang [21] proposed an empirical model to predict the value of *f*_*c*_′ of concrete with various SCMs based on a nonlinear multiple regression (NLMR) analysis using an extensive amount of test data collected from the available literature. In the database for the regression analysis, the primarily ranges of the main parameters are as follows: *W*/*B* = 0.25–0.6, *R*_*F*_ = 0.1–0.4, and *R*_*G*_ = 0.2–0.4. The number of ternary-type-binders using OPC, FA, and GGBS in the database is small and *R*_SCM_ is mostly within 0.5. Overall, the following equation, proposed by Yang, is thought to be suitable for concrete with a typical *R*_SCM_ not exceeding 0.5:

(1) $$\begin{array}{c} \frac{f_{c}^{\prime }}{f_{0}}=1.12{\left[W/B\left(1+R_{F}^{2}+R_{G}^{3}-R_{S}^{2}\right){\left(A_{c}\right)}^{0.1}\right]}^{-1.06}, \end{array}$$

where *f*_0_ (=10 MPa) is the reference value for the 28-day compressive strength of concrete and *R*_*S*_ is the silica fume level as a partial replacement for OPC.

**Figure 3:**
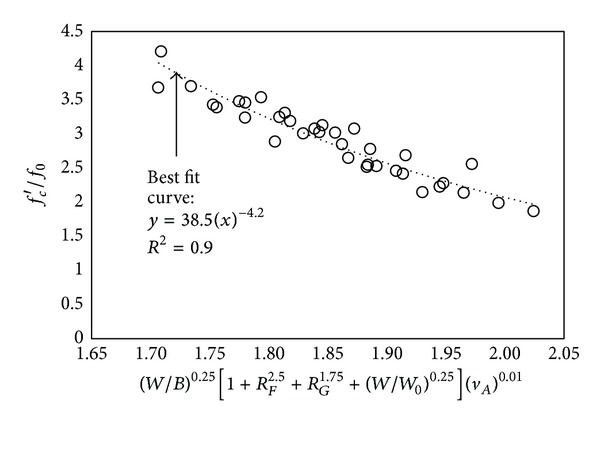
Regression analysis for *f*_*c*_′ of high-volume SCM concrete.

Table 4 clearly shows that *f*_*c*_′ of high-volume SCM concrete is somewhat sensitive to *W*, though sensitivity depends on the type and level of SCMs. Furthermore, to obtain the same *f*_*c*_′ of OPC concrete or concrete with a typical SCM level, a lower *W*/*B* is required for high-volume SCM concrete as compared to OPC concrete or typical SCM concrete. Considering these experimental observations, (1) was modified using the current test data to predict the *f*_*c*_′ of high-volume SCM concrete (see Figure 3). Consider

(2) fc′f0=38.5[(W/B)0.25     ×(1+RF2.5+RG1.75+(W/W0)0.25)(Ac)0.01]−4.2,

where *W*_0_ (=100 kg/m^3^) is the reference value for the unit water content.

**Figure 4:**
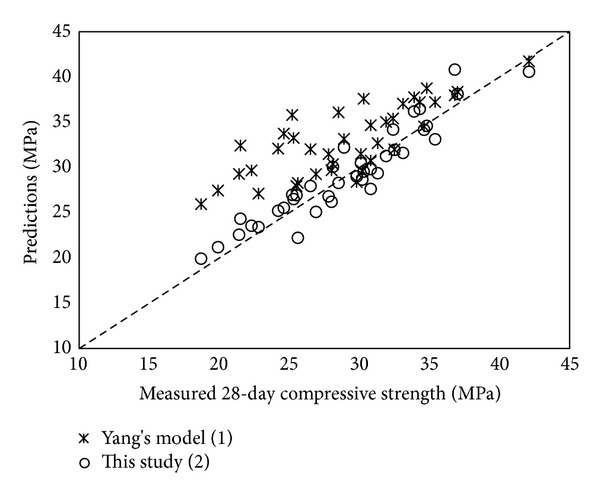
Comparisons of predicted and measured 28-day compressive strength.

Comparisons of the measured 28-day compressive strength and predictions obtained from the Yang's model (1) and the current model (2) are plotted in Figure 4. The current model gives lower values of *f*_*c*_′ than the Yang's model. The mean and standard deviation of the ratios between the experimental results and the predicted results are 0.89 and 0.103, respectively, for the Yang's model and 0.99 and 0.062 for the current model. This indicates that the Yang's model based on concrete mixes with typical SCM levels is likely to overestimate the 28-day compressive strength of high-volume SCM concrete.

### 3.3. Compressive Strength Development

**Figure 5:**
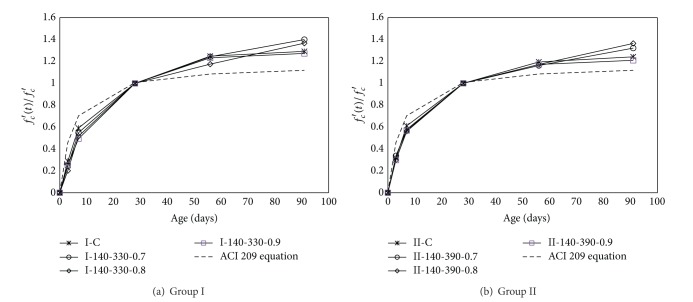
Typical compressive strength development rate of high-volume SCM concrete.

The typical compressive strength development rate of high-volume SCM concrete is shown in Figure 5. On the same figure, predictions determined from the ACI 209 equation [22] are plotted for comparison. It was difficult to determine the effect of *W* on the strength development rate. As *R*_SCM_ increased, the strength gain ratio at an early age relative to the 28-day strength tended to decrease, whereas that at a long-term age increased up to *R*_SCM_ of 0.8, beyond which it decreased somewhat. A slightly higher ratio at an early age and a slightly lower ratio at a long-term age were observed for Group II mixes as compared to Group I mixes, indicating that the strength development rate is affected by *W*/*B*. Relative to the 28-day strength of high-volume SCM concrete, the strength gain ratio at an age of 3 days ranged between 0.2 and 0.28 for Group I mixes and between 0.27 and 0.33 for Group II mixes, whereas that at age of 91 days ranged between 1.33 and 1.46 for Group I mixes and between 1.27 and 1.43 for Group II mixes. As compared with the predictions from the ACI equation, those values are lower by approximately 27–50% at 3 days and higher by approximately 14–31% at 91 days. This indicates that, by the ACI 209 equation, the compressive strength of very-high-volume SCM concrete is likely to be slightly overestimated at an early age or, conversely, underestimated at a long-term age. Although the specially modified polycarboxylate-based water-reducing agent was added to enhance the early strength of very high-volume SCM concrete, a strength gain lower than that found using the ACI 209 equation was measured at the ages of 3 and 7 days. However, it can be estimated that these low gains at an early age are not detrimental because the early strength gain of concrete with typical *R*_SCM_ is frequently found to be 10–40% lower than that of OPC concrete or the values predicted using the ACI 209 equation [7, 9]. Hence, the specially modified polycarboxylate-based water-reducing agent is expected to contribute to the early strength gain of very-high-volume SCM concrete.

**Figure 6:**
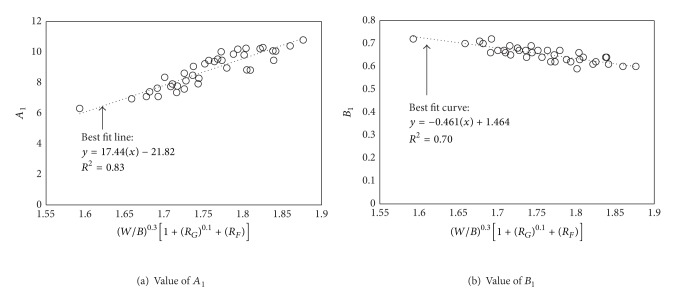
Regression analysis for constants *A*_1_ and *B*_1_ in (3).

The ACI 209 provision [22] empirically recommends the following parabolic strength development equation based on test results of OPC concrete:

(3) $$\begin{array}{c} f_{c}^{\prime }\left(t\right)=\frac{t}{A_{1}+B_{1}t}f_{c}^{\prime }, \end{array}$$

where *f*_*c*_′(*t*) is the compressive strength according to age *t* (in days). The strength development rate at early and long-term ages is determined by the variation of the constants *A*_1_ and *B*_1_. In general, a lower value of *A*_1_ leads to a higher compressive strength gain at an early age. For OPC concrete cured by air drying, it is recommended that the values of *A*_1_ and *B*_1_ are 4.0 and 0.85, respectively. However, these values need to be modified for very-high-volume SCM concrete in order to minimize the error observed in Figure 5. To fit the strength development characteristics of high-volume SCM concrete, the values of both constants were determined using test data (see Table 4). All specimens had a high correlation coefficient (*R* ^2^) of more than 0.93, as listed in Table 4. With the increase of the *W*/*B*, *R*_*G*_, and *R*_*F*_, the constant *A*_1_ tends to increase, whereas *B*_1_ decreases. The determined values of the constants appear to be more significantly affected by *R*_*G*_ than by *R*_*F*_, whereas they are independent of *W*. Based on regression analysis using these influencing parameters, the two constants *A*_1_ and *B*_1_ in (3) were proposed by the following linear equations (Figure 6):

(4) $$\begin{array}{c} A_{1}=17.44{\left(W/B\right)}^{0.3}\left(1+R_{G}^{0.1}+R_{F}\right)-21.82, \end{array}$$

(5) $$\begin{array}{c} B_{1}=-0.461{\left(W/B\right)}^{0.3}\left(1+R_{G}^{0.1}+R_{F}\right)+1.464. \end{array}$$

**Figure 7:**
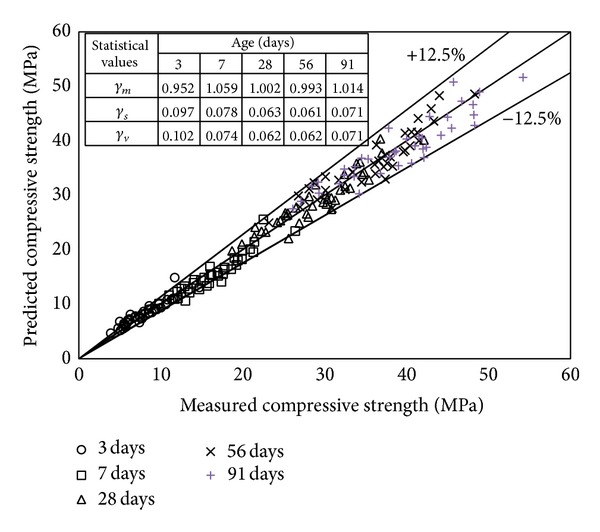
Comparison of predicted and measured strengths at different ages.

Comparisons of the measured and predicted compressive strengths at various ages are shown in Figure 7. Note that *f*_*c*_′ in (3) is determined using (2). The mean (*γ*_*m*_), standard deviation (*γ*_*s*_), and coefficient of variation (*γ*_*v*_) of the ratios between the experimental and predicted results are also given in the same figure. Compressive strengths at different ages predicted using (2)–(5) are mostly within ±12.5% of the measured values, giving values of *γ*_*m*_ and *γ*_*s*_ that range between 0.952 and 1.059 and between 0.061 and 0.097, respectively. The values of *γ*_*m*_ and *γ*_*s*_ for all tested ages were calculated to be 1.0 and 0.082, respectively. The proposed equations describe well the compressive strength development of very high-volume SCM concrete according to age.

### 3.4. Durability

**Figure 8:**
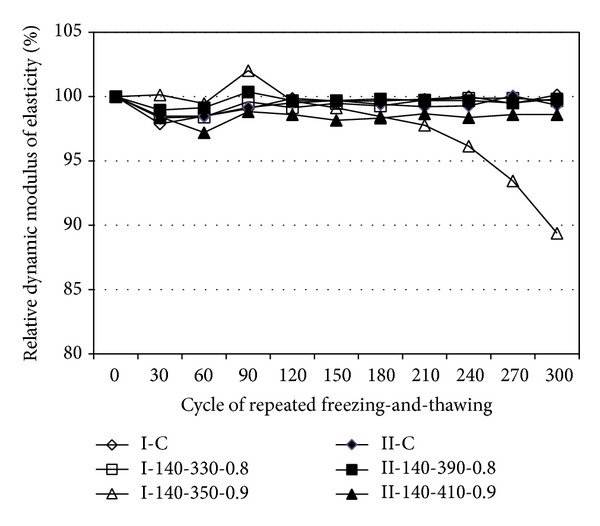
Freezing-and-thawing resistance of concrete tested.

The relative dynamic modulus of elasticity (*E*_*d*_) recorded every 30 cycles of freezing-and-thawing is shown in Figure 8. The control mixes maintained values of *E*_*d*_ above 98% throughout the 300 freezing-and-thawing cycles. The high-volume concrete with *R*_SCM_ of 0.8 showed the same behavior as the control mixes. For the very-high-volume concrete with *R*_SCM_ of 0.9, the value of *E*_*d*_ remained at 98% until the 210th freezing-and-thawing cycle, beyond which it gradually decreased to 90% until the end of the tests (300 cycles). This indicates that the freezing-and-thawing resistance of the selected high-volume SCM concrete mixes is comparable to that of the control mixes with the typical *R*_SCM_.

**Figure 9:**
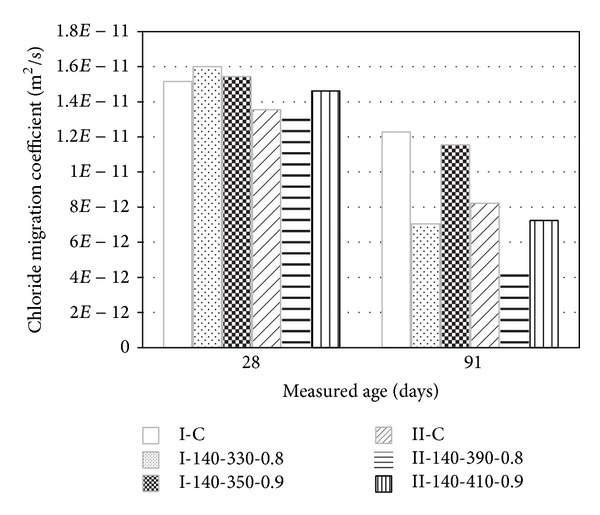
Chloride migration coefficient of concrete measured at 28 and 91 days.

Figure 9 presents the nonsteady-state chloride migration coefficients (*D*_nssm_) of concrete specimens at ages of 28 and 91 days, which were calculated from the measured chloride penetration depth, in accordance with the procedure specified in NT Build 492 [20]. As expected, the concrete with a designed strength of 30 MPa (Group II) had lower values of *D*_nssm_ than that with designed strength of 24 MPa (Group I). Furthermore, the value of *D*_nssm_ tended to decrease with increasing age. The ratios of *D*_nssm_ values between ages of 91 and 28 days were calculated to be 0.81 and 0.61 for the I-C and II-C control specimens, respectively, and 0.44 and 0.33 for the I-140-330-0.8 and II-140-390-0.8 specimens, indicating that the decrease of *D*_nssm_ with age is higher in the very-high-volume SCM concrete than in the control concrete. At an age of 28 days, a slightly higher *D*_nssm_ was calculated for the very-high-volume SCM concrete than for the control concrete, regardless of concrete compressive strength. However, at an age of 91 days, *D*_nssm_ tended to decrease with increasing *R*_SCM_ up to 0.8, beyond which it started to increase.

**Figure 10:**
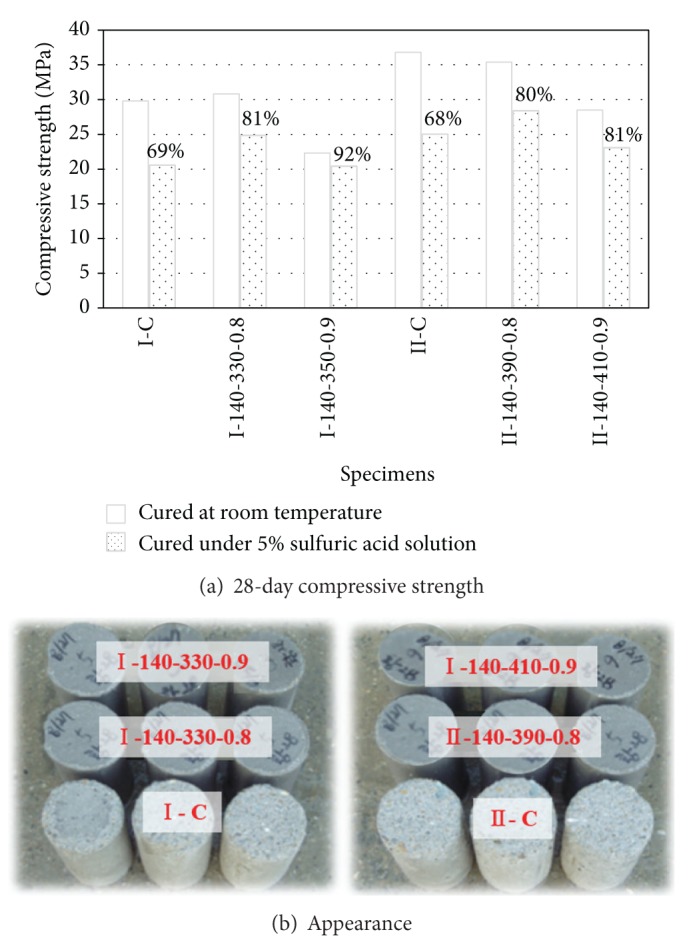
Variation of strength and appearance of concrete after saturation in sulfuric acid solution.

The variation of compressive strength of concrete measured from specimens saturated in 5% sulfuric acid solution for 28 days is shown in Figure 10(a). The appearance of those specimens is presented in Figure 10(b). The deterioration ratio of *f*_*c*_′ owing to the saturation in sulfuric acid solution was between 30 and 32% for the control concrete, whereas it decreased to 8–20% for the very-high-volume SCM concrete; in other words, the ratio of *f*_*c*_′ after saturation in a sulfuric acid solution for 28 days relative to the concrete cured at room temperature was measured to be 69%, 81%, and 92% for specimens I-C, I-140-330-0.8, and I-140-350-0.9, respectively. This indicates that the deterioration of *f*_*c*_′ owing to sulfate attack decreased with increasing *R*_SCM_. This trend was similarly observed in terms of the damage to the specimens; that is, the presence of damaged chips and flaws decreased with increasing *R*_SCM_. Hence, it can be proposed that the very-high-volume SCM concrete has superior sulfate resistance as compared to conventional concrete.

The beneficial effect of SCMs on the durability of concrete can be explained by improvement in both the impermeability and diffusion taking place in water-filled pores or by capillary suction. Gruyaert et al. [23] showed that the value of *D*_nssm_ in concrete mixes with *R*_*G*_ varying from 0 to 0.85 recorded at an age of 91 days decreases with increasing *R*_*G*_. However, the addition of SCM exceeding a certain limit would result in decreased impermeability, as demonstrated in freezing-and-thawing and chloride resistances. Hence, *R*_SCM_ needs to be restricted to less than 0.8–0.85 in order to maintain a positive influence on the durability of concrete.

## 4. Conclusions

The present investigation needs to be further extended to examine the carbonation resistance and inelastic deformation of very-high-volume SCM concrete in order to improve the compressive strength. From the experimental observations on the compressive strength and durability in the current study, the following conclusions may be drawn.

1. The compressive strength of the concrete with *W* of 150 kg/m^3^ was commonly lower than that of the companion concrete with *W* of 140 kg/m^3^ by approximately 10%, even at the same *W*/*B*, showing that *f*_*c*_′ of high-volume SCM concrete is somewhat sensitive to *W*.
2. To achieve a value of *f*_*c*_′ equivalent to that of conventional concrete with typical *R*_SCM_, *W*/*B* and *R*_SCM_ in very-high-volume SCM concrete need to be restricted to less than 40% and to 0.7, respectively.
3. As *R*_SCM_ increased, the strength gain ratio at an early age relative to the 28-day strength tended to decrease, whereas that at a long-term age increased until reaching *R*_SCM_ of 0.8, beyond which it decreased somewhat.
4. Unlike the ACI 209 equation which overestimates the early strength of high-volume SCM concrete and underestimates the strength at a long-term age, the proposed equations describe well the compressive strength development of very-high-volume SCM concrete; the mean and standard deviations of the ratios between the experimental and predicted results were 1.0 and 0.082, respectively.
5. In general, the freezing-and-thawing, chloride, and sulfate resistances of the high-volume SCM concrete mixes were comparable to those of the control mixes with the typical *R*_SCM_. However, the beneficial effect of SCMs on the freezing-and-thawing and chloride resistances of concrete decreased at *R*_SCM_ of 0.9.

## Figures and Tables

**Table 1:** Chemical composition of the cementitious materials (% by mass).

Materials	SiO_2_	Al_2_O_3_	Fe_2_O_3_	CaO	MgO	K_2_O	Na_2_O	TiO_2_	SO_3_	LOI∗
OPC	22.1	5.0	3.0	64.8	1.6	0.54	0.35	0.30	2.0	0.31
FA	53.3	27.9	7.8	6.79	1.11	0.84	0.55	—	0.82	0.89
GGBS	31.55	13.79	0.53	44.38	5.2	0.4	0.18	0.98	2.79	0.2

*Loss on ignition.

**Table 2:** Physical properties of aggregates used.

Type	Maximum size (mm)	Unit volume weight (kg/m^3^)	Specific gravity	Water absorption (%)	Porosity (%)	Fineness
Coarse particles	25	1447	2.62	1.78	43.22	7.05
Fine particles	5	1566	2.61	1.16	33.51	2.83

**Table 3:** Designed properties and main parameters of concrete specimens.

Type	Designed properties			Test parameters				
	*f*_*cu*_ (MPa)	*A*_*c*_ (%)	*S*_*i*_ (mm)	*R*_*F*_	*R*_*G*_	*R*_SCM_	*B* (kg/m^3^)	*W* (kg/m^3^)
Control mix	24	4.5 ± 1.5	210 ± 25	0.25	0.15	0.4	342	184
	30						400	165
Very high-volume SCM mix	24 30			0.4	0.3 0.4 0.5	0.7 0.8 0.9	310 330 350 370 390 410	140 150

*f*_*cu*_: Designed compressive strength of concrete at age of 28 days, *A*_*c*_: air content of fresh concrete, *S*_*i*_: initial slump of fresh concrete, *S*/*a*: fine aggregate-to-total aggregate ratio by volume, *R*_*F*_: FA level for partial replacement of OPC, *R*_*G*_: GGBS level for partial replacement of OPC, *R*_SCM_: total SCM level for partial replacement of OPC, *B*: unit binder content, and *W*: unit water content.

**Table 4:** Details of concrete mixture proportions and summary of test results.

Specimen	*W*/*B* (%)	*S*/*a* (%)	*B* (kg/m^3^)	Unit weight (kg/m^3^)								Test result							Determination of constants in (3)		
				*W*	*C*	FA	GGBS	*S*	*G*	*R*_*A*_ (%)	*R*_*SP*_ (%)	*A*_*c*_ (%)	*S*_*i*_ (mm)	*f*_*c*_′ (MPa) at different ages (days)							
														3	7	28	56	91	*A*_1_	*B*_1_	*R* ^2^
I-C	53.8	48	342	184	205	86	51	816	888	0.025	0.50	4.0	205	8.6	17.6	29.8	37.2	38.5	7.59	0.68	0.99
I-140-310-0.7	45.1		310	140	93	124	93	877	953	0.032	0.85	4.2	210	7.4	15.3	28.1	38	40	9.82	0.59	0.99
I-140-310-0.8	45.1		310	140	62	124	124	875	952	0.032	0.85	3.8	205	5.9	14.7	28	34.5	39	10.23	0.61	0.99
I-140-310-0.9	45.1		310	140	31	124	155	875	951	0.032	0.75	3.7	200	5.4	13	25.6	30.9	34.2	10.09	0.64	0.99
I-140-330-0.7	42.4		330	140	99	132	99	867	943	0.032	0.85	4.8	205	7.9	17.8	32.5	40.4	45.5	9.55	0.62	0.99
I-140-330-0.8	42.4		330	140	66	132	132	866	942	0.032	0.80	5.1	215	6.2	15.9	30.8	36.2	42.1	9.87	0.63	0.99
I-140-330-0.9	42.4		330	140	33	132	165	865	940	0.036	0.80	4.4	205	5.6	11	22.3	27.5	28.4	10.26	0.66	0.99
I-140-350-0.7	40		350	140	105	140	105	858	933	0.032	0.85	4.2	220	8.6	19.4	34.6	41.3	48.2	9.07	0.64	0.99
I-140-350-0.8	40		350	140	70	140	140	856	931	0.032	0.75	5.2	210	6.3	17	31.3	37.6	42.4	9.46	0.64	0.99
I-140-350-0.9	40		350	140	35	140	175	855	930	0.036	0.80	3.8	215	5	12.5	24.2	30.1	33.6	10.03	0.65	0.99
I-150-310-0.7	48.3		310	150	93	124	93	864	940	0.042	0.70	4.6	215	7.4	13.3	25.5	30.1	35.3	9.47	0.64	0.99
I-150-310-0.8	48.3		310	150	62	124	124	863	938	0.042	0.70	4.5	215	4.8	11.7	22.8	29.1	31.8	10.41	0.6	0.99
I-150-310-0.9	48.3		310	150	31	124	155	862	937	0.042	0.70	4.2	200	3.9	9.3	18.7	23.2	26.1	10.8	0.6	0.99
I-150-330-0.7	45.4		330	150	99	132	99	855	929	0.042	0.75	5.0	210	7.7	17.4	30.2	38.3	42	8.84	0.63	0.99
I-150-330-0.8	45.4		330	150	66	132	132	853	928	0.038	0.70	4.1	205	5.6	13.5	26.9	32.5	36.8	10.31	0.62	0.99
I-150-330-0.9	45.4		330	150	33	132	165	852	927	0.042	0.70	4.6	215	5.1	9.8	19.9	25.5	27.1	10.07	0.61	0.99
I-150-350-0.7	42.8		350	150	105	140	105	845	919	0.030	0.70	5.0	215	7.7	16.1	30.1	37.4	41.9	9.47	0.62	0.99
I-150-350-0.8	42.8		350	150	70	140	140	844	918	0.028	0.65	3.7	210	6.1	15.6	27.8	37.4	40.6	10.2	0.62	0.99
I-150-350-0.9	42.8		350	150	35	140	175	843	916	0.042	0.65	4.5	215	5.7	12.1	21.4	27	29.3	8.83	0.64	0.99

II-C	41.2	46	400	165	240	100	60	814	886	0.035	0.80	3.7	210	11.7	22.5	36.8	44	45.7	6.32	0.72	0.99
II-140-370-0.7	37.8		370	140	111	148	111	812	958	0.032	0.85	3.6	220	11.3	20	34.3	41.4	46.7	7.75	0.67	0.99
II-140-370-0.8	37.8		370	140	74	148	148	812	956	0.032	0.80	4.7	230	9.5	17.8	31.9	39.6	41.5	8.13	0.67	0.99
II-140-370-0.9	37.8		370	140	37	148	185	810	955	0.036	0.70	4.7	225	7.5	14	25.3	28.7	32.4	7.93	0.69	0.99
II-140-390-0.7	35.8		390	140	117	156	117	804	947	0.032	0.85	4.6	230	12.5	21.4	37	43	48.9	7.41	0.7	0.99
II-140-390-0.8	35.8		390	140	78	156	156	802	945	0.032	0.75	4.5	220	10.6	19.9	35.4	41.5	48.3	8.36	0.67	0.99
II-140-390-0.9	35.8		390	140	39	156	195	801	944	0.036	0.70	3.7	210	8.5	16.2	28.5	33.4	34.5	7.36	0.69	0.99
II-140-410-0.7	34.2		410	140	122.3	164.4	123.3	794	936	0.034	0.80	3.3	210	14.7	26.4	42.1	48.3	54.2	6.95	0.7	0.99
II-140-410-0.8	34.2		410	140	81.2	164.4	164.4	794	935	0.036	0.80	4.9	235	10.8	21.3	34.8	40.6	45	7.1	0.71	0.99
II-140-410-0.9	34.2		410	140	40.1	164.4	205.5	792	933	0.035	0.70	4.0	225	9.2	18.6	30.3	36.1	38.7	7.1	0.72	0.99
II-150-370-0.7	40.5		370	150	111	148	111	800	943	0.042	0.70	5.4	215	8.5	16	28.9	36.3	37.8	8.29	0.66	0.99
II-150-370-0.8	40.5		370	150	74	148	148	800	942	0.040	0.65	5.5	220	6.9	14.9	26.5	33.4	37.9	9.39	0.67	0.99
II-150-370-0.9	40.5		370	150	37	148	185	798	940	0.040	1.00	3.0	220	5.5	12	21.5	26.8	29.2	8.97	0.67	0.99
II-150-390-0.7	38.4		390	150	117	156	117	792	933	0.045	1.00	4.9	230	9.8	19	32.4	39.8	42.8	7.78	0.65	0.99
II-150-390-0.8	38.4		390	150	78	156	156	790	931	0.045	0.90	4.4	220	8.9	16.9	30.8	36.6	41	8.49	0.67	0.99
II-150-390-0.9	38.4		390	150	39	156	195	788	930	0.045	0.90	3.5	210	6	12.9	24.6	28.1	32.4	9.24	0.67	0.99
II-150-410-0.7	36.5		410	150	123	164	123	782	922	0.045	0.90	4.4	215	11.5	20.9	33.9	43.3	48.1	7.64	0.66	0.98
II-150-410-0.8	36.5		410	150	82	164	164	780	920	0.045	0.90	3.7	220	10	19.2	33.1	39.8	44.2	7.92	0.66	0.99
II-150-410-0.9	36.5		410	150	41	164	205	780	919	0.045	0.90	3.3	220	6.9	14.1	25.2	31.7	33.7	8.62	0.68	0.98

## Notations

*A*_*c*_: Initial air content of fresh concrete
*B*: Unit binder content
*D*_nssm_: Nonsteady state chloride migration coefficient
*E*_*d*_: Relative dynamic modulus of elasticity
*G*: Unit coarse aggregate content
*f*_*c*_′: Measured concrete compressive strength at an age of 28 days
*f*_0_: Reference concrete compressive strength (=10 MPa)
*f*_*c*_′(*t*): Concrete compressive strength at age *t* (in days)
*f*_*cu*_: Designed 28-day compressive strength of concrete
*R*_*A*_: Ratio of air entraining agent to binder by weight
*R*_*F*_: Ratio of fly ash to binder by weight
*R*_*G*_: Ratio of granulated ground blast-furnace slag (GGBS) to binder by weight
*R*_*S*_: Ratio of silica fume (SF) to binder by weight
*R*_SCM_: Ratio of supplementary cementitious materials (SCMs) to binder by weight
*R*_*SP*_: Ratio of the modified polycarboxylate-based water-reducing agent to binder by weight
*S*: Unit fine aggregate content
*S*/*a*: Fine aggregate-to-total aggregate ratio by volume
*S*_*i*_: Initial slump of fresh concrete
*W*: Unit water content
*W*_0_: Reference value for the unit water content (=100 kg/m^3^)
*W*/*B*: Water-to-binder ratio
*γ*_*m*_: Mean of the ratios (*γ*_*cs*_) between experiments and predicted compressive strengths
*γ*_*s*_: Standard deviation of *γ*_*cs*_
*γ*_*v*_: Coefficient of variation of *γ*_*cs*_.
